# Genetic and Biological Basis of High Royal Jelly-Producing Bees *Apis mellifera ligustica*: From Larval Signal Perception to Glandular Secretion

**DOI:** 10.3390/insects17101065

**Published:** 2026-10-15

**Authors:** Na Wu, Weiran Ao, Zhiping Li, Jianke Li

**Affiliations:** 1 Beijing Houdeli Bee-Products Company, East Dajiaoting Middle Street, Beijing 100124, China; 2 Institute of Apicultural Research, Chinese Academy of Agricultural Sciences, Yumingyuanxi Road, Beijing 100191, China

**Keywords:** royal jelly, *Apis mellifera ligustica*, artificial selection, hypopharyngeal gland, 10-HDA, larval pheromone, olfaction, neuropeptidome

## Abstract

Royal jelly (RJ), the glandular secretion that determines honeybee caste, is also a high-value apicultural product. China supplies about 90% of the world production volume, almost entirely through high RJ-producing bees (RJBs) selected from *Apis mellifera ligustica* over four decades, or Chinese-selected *a. m. ligustica* population; these yield up to ten times more RJ per colony than ITBs. Here, we review and assemble the morphological, proteomic, genomic and behavioural evidence for that unusual selection response at four levels. RJ production may be dominated by a heritable polygenic trait whose acceptance and secretion components carry significant genotypic variance, and the selected candidate genomic architecture combines a large inversion with the tandem *mrjp* cluster. RJB nurses could detect larval volatiles more sensitively, and cross-fostering suggests that larval acceptance depends on the genotype of the nurse bees, not that of the larvae. RJB hypopharyngeal glands develop larger acini, delayed regression and reinforced protein synthesis, whereas mandibular glands up-regulate the fatty-acid pathway that may yield 10-HDA. An expanded nurse-stage neuropeptidome and a remodelled brain membrane proteome may tune larval recognition, water homeostasis and pollen collection. The proposed three coupled feedback loops and outline the experiments needed to test the model.

## Simple Summary

Royal jelly is the protein-rich food that turns a honeybee larva into a queen, and it is also one of the most valuable products of beekeeping. Over the past several decades, Chinese beekeepers have bred high royal jelly-producing bees (RJBs) that yield roughly ten times more royal jelly than unselected Italian bees (ITBs). This review explains how they achieve it. The antennae of RJBs may sense the chemical signals of young larvae more keenly, so their nurses accept and feed more of them. The hypopharyngeal glands of RJBs are larger, stay active for longer, and build protein faster. The mandibular gland makes more of the fatty acid that marks jelly quality. The brain chemistry governing nursing and pollen gathering has shifted to match. None of this required a new organ or a new kind of sensor; the changes lie in sensitivity, capacity and timing. Understanding them helps breeders choose better honeybee stocks, and indicates how human selection can reshape a honeybee line while leaving its basic body plan intact.

## 1. Introduction

Royal jelly (RJ) is a milky, protein-rich secretion of the hypopharyngeal and mandibular glands of young worker honeybees. Every female larva receives it for the first three days and the queen-destined larva exclusively thereafter, so RJ decides whether a larva develops into a queen or a worker [1,2]. It is at the same time a high-value product: its major royal jelly proteins (MRJPs), its signature fatty acid 10-hydroxy-2-decenoic acid (10-HDA) and its peptides carry antibacterial, antioxidant and immunomodulatory activity. Increasing RJ production to satisfy human need is a long pursuit for beekeepers and scientists.

China now dominates world RJ production, and that position rests on a single breeding achievement of RJBs. Annual output climbed from roughly 200 t in the 1980s to about 3000 t today, with China supplying more than 90% of the global production volume [3,4]. Beginning in the 1950s, beekeepers in Zhejiang and Jiangsu found colonies of the Italian honeybee *Apis mellifera ligustica* that happened to produce unusually large amounts of RJ [4]. The resulting RJBs now yield more than 10 kg of RJ per colony per year, against a few hundred grams in ITBs of the same subspecies [3,5].

**Figure 1:**
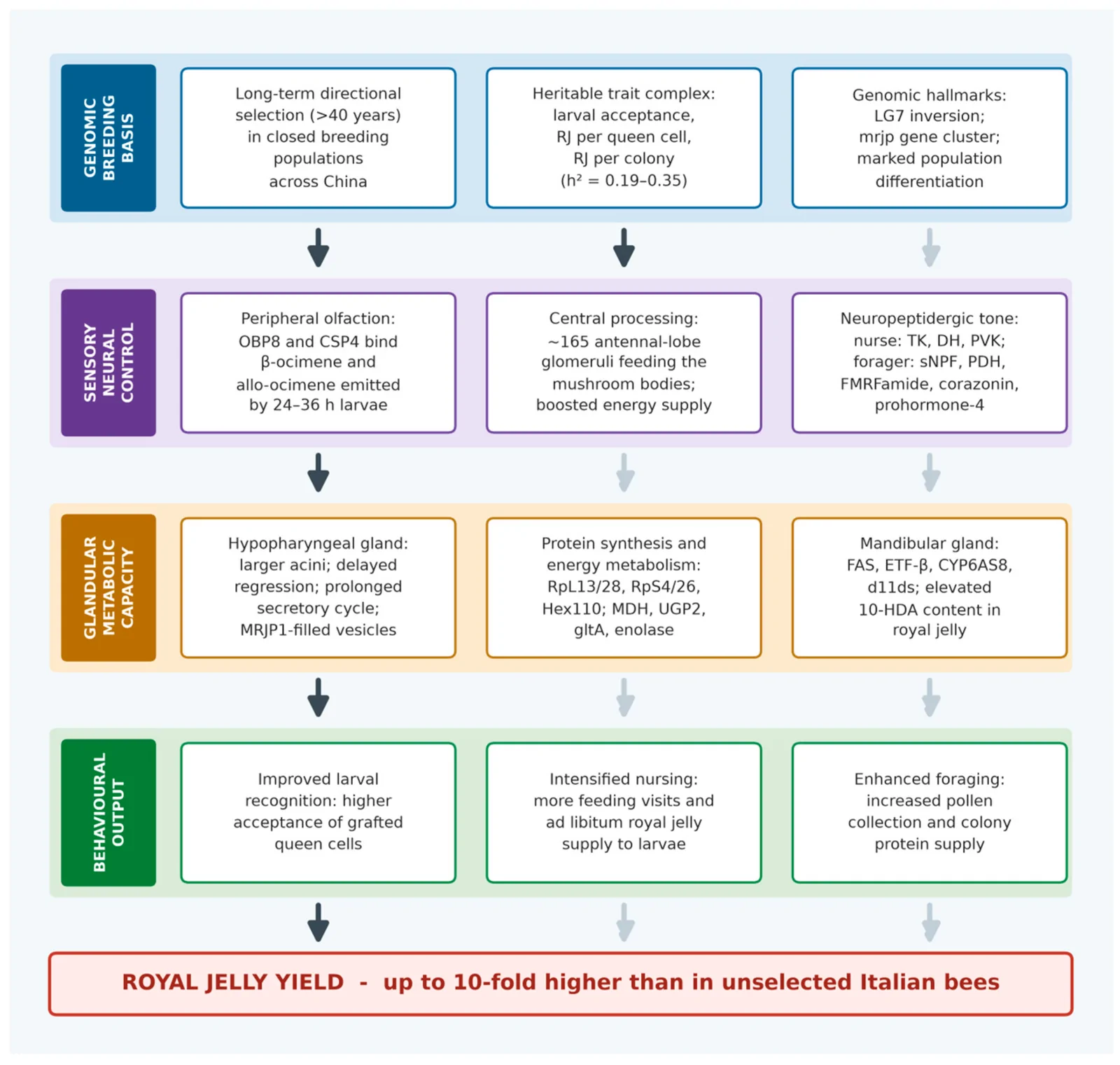
The proposed integrated framework linking the genetic, sensory, glandular and neural layers that together may determine royal jelly yield. The figure is drawn based on the published data in this review, in which the solid arrows are experimentally demonstrated, and grey arrows are from omics data.

Accounting for a response of this production size requires evidence from several biological levels at once, because RJ output depends on how many larvae a colony accepts, how much secretion each accepted cell receives, and how much protein the colony can route to its nurses. Work on the RJBs now spans that range, from a chromosome-scale genome assembly to neuropeptidomics of the worker brain (Figure 1) [4,6]. What it has not yet had is a single synthesis, and it remains unclear which of the documented changes are causes and which are downstream consequences of heavier secretion.

This review takes up that synthesis. It begins with the genetic architecture and breeding history of the stock, then treats the hypopharyngeal gland, the mandibular gland and 10-HDA synthesis, the olfactory recognition of larval signals, and the neural control of nursing and foraging. It closes with an integrated model of three coupled loops and the experiments that would test it.

## 2. Genetic Architecture and Breeding History of the RJBs

Four decades of selection have produced a stock of RJBs whose RJ yield is several times that of ITBs, yet the genomic basis of the response is only beginning to come into focus. What is already well established is that the trait is heritable and polygenic in the production performance of the RJBs.

### 2.1. Domestication and the Selection Response

**Figure 2:**
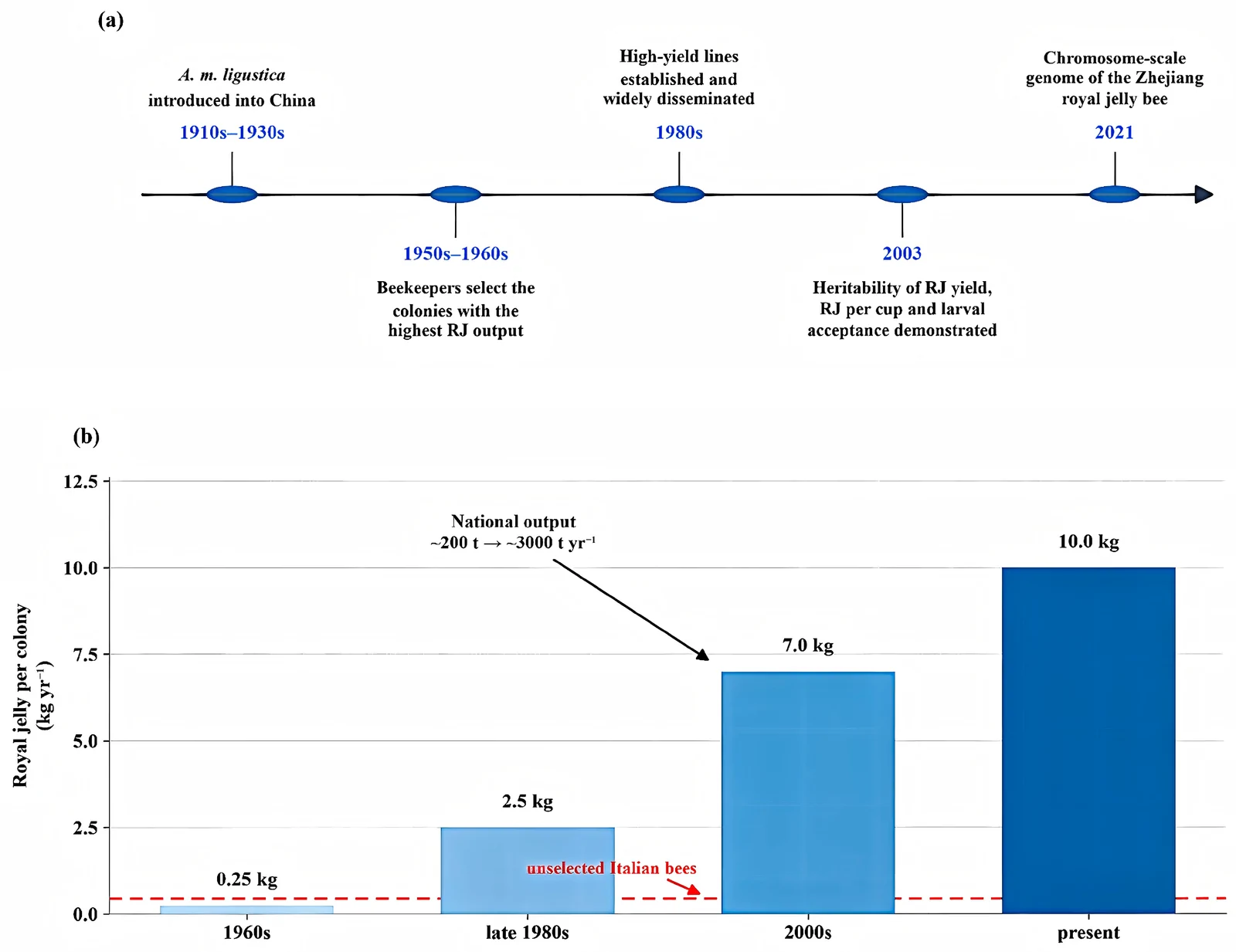
(**a**) Timeline of the introduction of *Apis mellifera ligustica* into China and of selecting high-yield royal jelly bees. (**b**) Reported annual yield per colony from the 1960s to the present; the red dashed line is royal jelly production of unselected Italian bees, the blue histogram is royal jelly yield of high-yield royal jelly bees. The figure is redrawn from published values [3,4,7].

The stock arose through directional selection within the introduced ITBs that were introduced to China in the 1910s and 1930s and became the dominant managed honeybee of the eastern provinces. RJ harvesting expanded through the 1950s and 1960s, and beekeepers began breeding from whichever colonies yielded most [4]. Selection started as mass selection within apiaries, was extended by exchanging queens between high-yielding RJ colonies, and matured into closed group propagation. The RJBs are the best documented product of that process (Figure 2) [4,7]. Reported annual yield per colony rose from 0.2 to 0.3 kg in the 1960s to 2–3 kg in the late 1980s, 6–8 kg in the 2000s, and more than 10 kg at present [3,4].

### 2.2. Quantitative Genetics of Royal Jelly Production

The first genetic investigation of the heritability of the trait came from a developmental genetic analysis of colony RJ yield, RJ per queen cell and the acceptance rate of queen cell cups across three lines of western honeybees: ITBs, native ITBs that the queens are kindly offered from Italian beekeepers, and Chinese ITBs that are not selected for enhanced RJ yields [8]. All colonies headed by three lines of honeybees with equalized colony structure and queen age are maintained in the same apiary to minimize the environmental influence. Thus, we tentatively conclude that genotypic variance is significant for colony yield and yield per cup at every stage, and for cup acceptance at ten of eleven stages, so all three components are predominantly genetically determined. The same analysis suggests conditional gene expression that may switch on only at particular stages of the harvesting period [8]. Genetic correlations between stages are significant for yield and yield per cup, though not consistently for cup acceptance, indicating that early-acting genetic effects propagate forward into later performance [8].

Independent estimates from the French RJ breeding program agree. Under a queen model, heritability was 0.19 ± 0.09 at the first harvest and 0.35 ± 0.13 at the second, with a genetic gain of about 3.5% per year [9]. Because the components are genetically correlated, they can be improved together; the polygenic, colony-level nature of the trait nonetheless means that reliable evaluation demands large datasets and careful treatment of mating structure [10].

Heritability is dependent on both population and environment. The two aforementioned studies investigated colony-level rather than individual-worker royal jelly production traits. This trait is affected by worker genotype, queen genotype, mating structure, colony environment, nutrition, brood composition and management practices. Although genetic factors may potentially play a dominant role in this trait, genotype–environment interactions or differences in nursing status, rather than genetic effects alone, still need to be treated with caution.

### 2.3. Morphological and Molecular Markers

Direct measurement of RJ yield is laborious, which has motivated the search for morphological, microsatellite and protein markers as surrogates. Among morphological traits, hypopharyngeal gland length shows the strongest correlation with yield per kilogram of bees, followed by acinar number, and it is recommended as a potential selection marker [7]. Microsatellite surveys reported the first molecular evidence of genetic differentiation: ten loci amplified 96 alleles across the RJBs, the ITBs and local Italian bees; seven alleles at six loci increased in frequency in the order ITBs < local Italian bees < RJBs, suggesting these loci are candidate molecular markers for yield [11]. Among protein markers, length polymorphism in the repetitive region of *mrjp3* may be a candidate queen mother marker in breeding programs [12].

### 2.4. Genomic Architecture and Its Limits

A chromosome-scale genome assembly of RJBs, constructed using PacBio long reads and Hi-C sequencing data, was recently released. The assembled genome spans 222 Mb distributed across 16 pseudo-chromosomes, with a scaffold N50 of 13.6 Mb and a BUSCO completeness of 99.3% [4]. It contains 12,288 predicted protein-coding genes, with repetitive sequences accounting for 8.11% of the genome. These values are close to those of the ITB genome, indicating that genome size, gene number and repeat content may not explain the high-yield RJ phenotype. One conspicuous structural difference between the two genomes is a large inversion in the RJB genome. Since inversions suppress local recombination and maintain co-adapted alleles as a single unit, this inverted region is considered a plausible contributor to the selected phenotype [4], though this hypothesis requires further experimental validation.

Mitochondrial genomes point elsewhere. Among 100 RJBs and 30 ITBs, an RJB-dominant sublineage is six times more frequent, but the pattern is attributable mainly to genetic drift rather than to positive selection, and mitogenome copy number was unrelated to RJ output [13].

Population-genomic scans have identified candidate regions, but causal mapping is inherently difficult in this species: colony-level phenotypes are produced by thousands of workers, and honeybee queens are both polyandrous and haplodiploid. Simulation studies show that quantitative trait locus discovery requires approximately 1000 samples with genotyping performed on both queens and workers [14]. Overall, current genetic evidence indicates a polygenic architecture: a small number of structural features—including the chromosome-scale inversion and the *mrjp* cluster—are situated against a broad background of small-effect regulatory variants [4,15].

## 3. The Hypopharyngeal Gland: Enlarged Acini, Extended Secretion and Reinforced Protein Synthesis

RJ protein is synthesized almost exclusively in the hypopharyngeal gland, and this is precisely the organ where selection has left its most consistent marks—these marks are reflected in gland size, the duration of the secretory phase, and the protein-synthesis machinery itself.

### 3.1. Structure and the Age-Dependent Secretory Cycle

**Figure 3:**
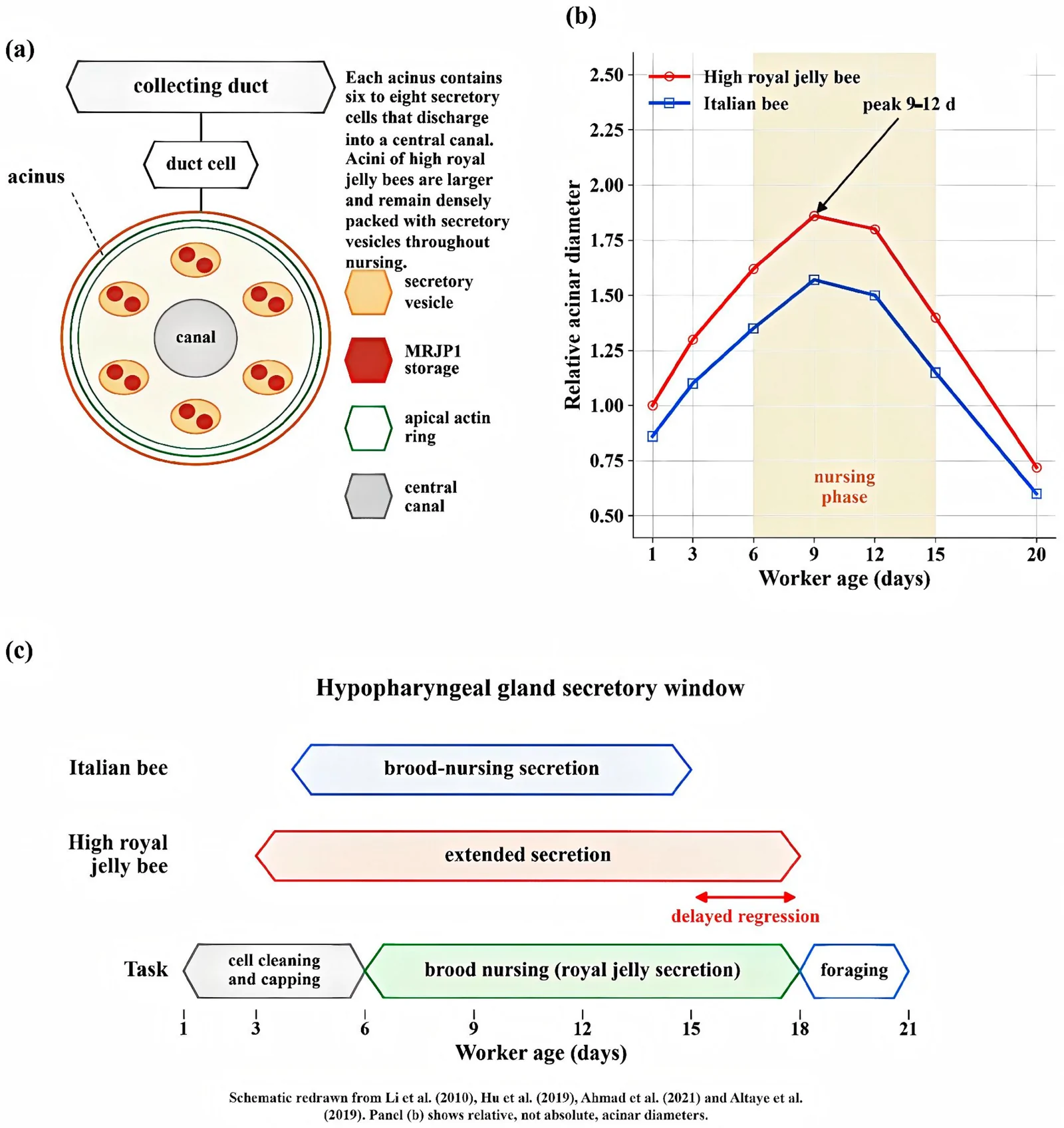
(**a**) Structure of a single hypopharyngeal acinus. (**b**) Acinar diameter across worker age in RJBs and Italian bees. (**c**) Secretory window relative to the behavioural task schedule [3,5,16,19]. Figure drawn de novo for this review has no graphical reconstruction that unintentionally implies numerical relationships absent from the original studies.

The hypopharyngeal gland is a paired tubular organ at the front of the worker head. Each secretory unit, or acinus, consists of six to eight secretory cells arranged around a central canal that drains through a duct cell into a collecting duct (Figure 3a) [16].

Gland activity follows the age-dependent division of labour, but feeding behaviour, rather than age, is the decisive stimulus. Acini grow after emergence, peak between days 6 and 15 in summer, then regress as the bee becomes a forager, at which point secretion switches from MRJPs to carbohydrate-active enzymes such as alpha-glucosidase and amylase [16,17]. Because both protein supply and brood signals shift the timing of this cycle, the gland is best understood as a plastic organ whose state follows colony demand [16,18].

### 3.2. Acinar Enlargement and Its Relation to Yield

Electron microscopy of the two stocks established the central morphological fact of the syndrome: RJB acini are significantly larger than those of ITBs at every age, and acinar diameter in both stocks fully develops until about day 12 before declining [5]. In the same study, RJBs produced roughly five times more RJ, and acinar size correlated positively with yield. These phenotype data suggest that it may be associated with the enhanced RJ production since a larger acinus holds more secretory cells and a larger vesicular reservoir per cycle, so each accepted cell can be provisioned faster (Figure 3b) [3,5].

### 3.3. Prolonged Secretion and Delayed Gland Regression

Comparative proteome studies reveal that the secretory period may be longer in RJBs, which therefore deliver RJ across a greater part of the nursing phase rather than only at its peak [3,5]. Because RJ is harvested over a fixed window, a longer competent period increases the number of secretion cycles a colony can complete (Figure 3c). The timing of regression is not fixed either: treated nurse bees can revert to nursing and the gland responds accordingly, so the extended window is a regulatory property, not a fixed structural one [19]. However, this proteome data demands further biological validation.

### 3.4. Vesicle Content, the Cytoskeleton and Protein Synthesis

Vesicle loading and the discharge machinery support rapid delivery. During the nursing phase, the secretory vesicles are densely packed with MRJP1, and actin rings of F-actin at the apical pole of each cell govern discharge into the canal [19].

**Figure 4:**
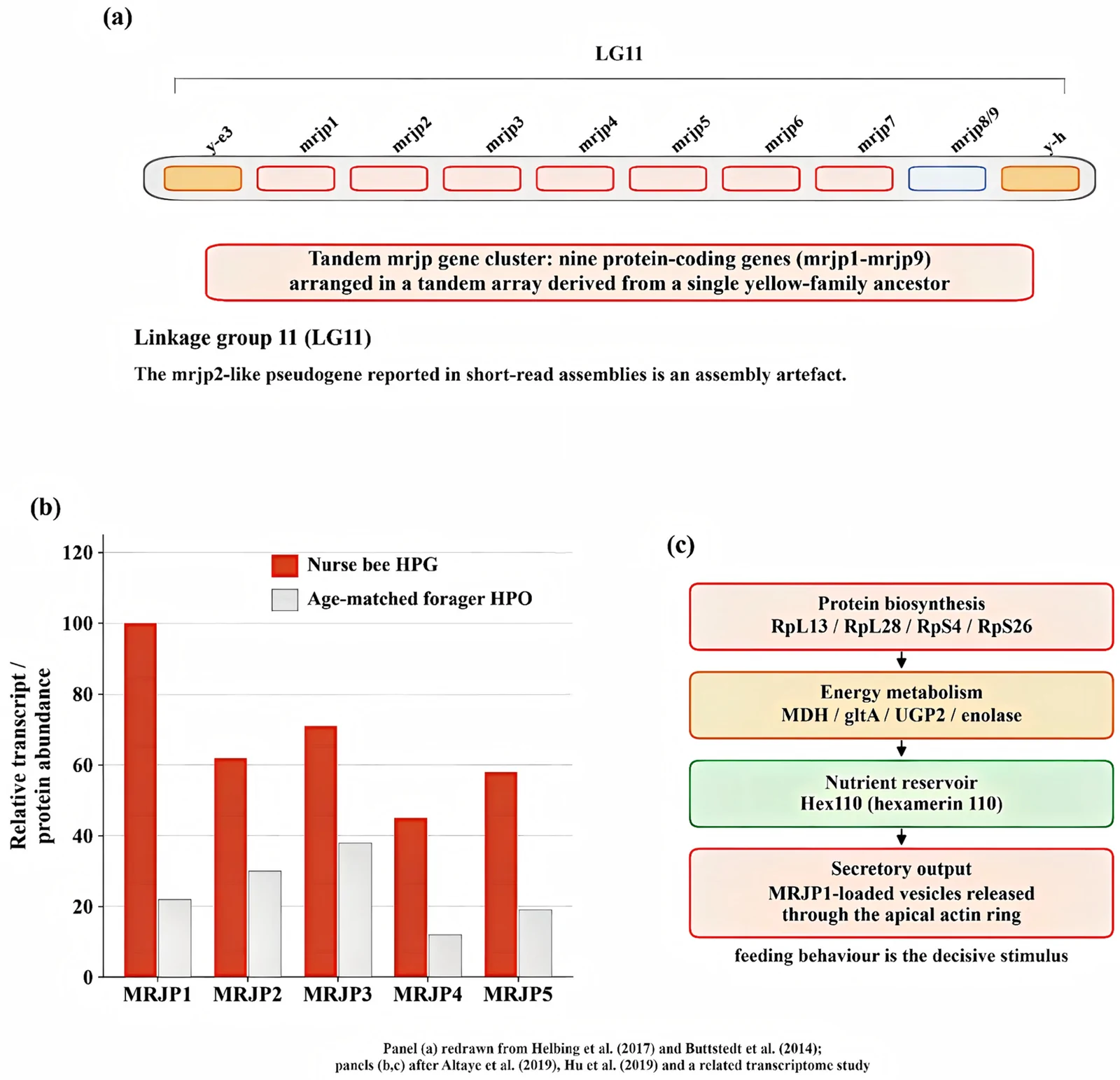
(**a**) The *mrjp* gene cluster on linkage group 11 [15,20]. (**b**) Abundance of MRJP1 to MRJP5 in nurse and forager glands. (**c**) The induced protein-synthesis and energy-metabolism network [3,19]. Figure drawn de novo for this review has no graphical reconstruction that unintentionally implies numerical relationships absent from the original studies.

Proteomics spanning the whole worker lifespan identify reinforced protein synthesis and energy metabolism as the functional signature of the RJBs. In RJB nurses, both pathways are induced relative to ITB nurses, including the ribosomal proteins RpL13, RpL28, RpS4 and RpS26, the storage protein hexamerin 110, and the energy-metabolism enzymes malate dehydrogenase, UGP2, citrate synthase and enolase (Figure 4c) [19]. Behavioural manipulation confirmed causality: expression of exactly these proteins rose when RJB nurses were recruited to nursing by altering colony composition. The response is also quantitative. Because protein concentration per unit RJ is preserved while output rises roughly tenfold, the synthesis machinery must scale in proportion, and the proteomic data indicate that selection has achieved precisely this [3,19].

### 3.5. The mrjp Gene Family and Its Expression

The MRJPs account for 80–90% of RJ protein and are encoded by a tandem array of nine genes, *mrjp1* to *mrjp9*, on linkage group 11 between the yellow-family genes *y-e3* and *y-h* (Figure 4a) [12,15]. All MRJPs descend from a single yellow-family ancestor, and the cluster expanded by tandem duplication. A *mrjp2*-like sequence reported as a pseudogene in short-read assemblies proved to be an artefact of the highly similar neighbouring genes [15].

Expression is tissue-biased but not gland-restricted. *mrjp1* to *mrjp7* are expressed predominantly in worker heads, with the strongest signal in nurses that have active glands, and MRJP1, MRJP4 and MRJP5 are markedly more abundant in nurse glands than in age-matched forager glands (Figure 4b) [5,20]. MRJP1, also known as royalactin, is the most abundant protein of RJ, but its proposed role as the sole queen-determining factor has been contested, and the current view emphasizes nutritional rather than instructive functions [21]. Several MRJPs also occur outside the gland, including in the brain, which bears on the neural arguments below (Table 1) [20,22].

## 4. The Mandibular Gland and the Biosynthesis of 10-HDA

The lipid fraction of RJ comes from the mandibular gland, whose principal product, 10-HDA, doubles as the standard marker of jelly quality.

### 4.1. The Pathway

**Figure 5:**
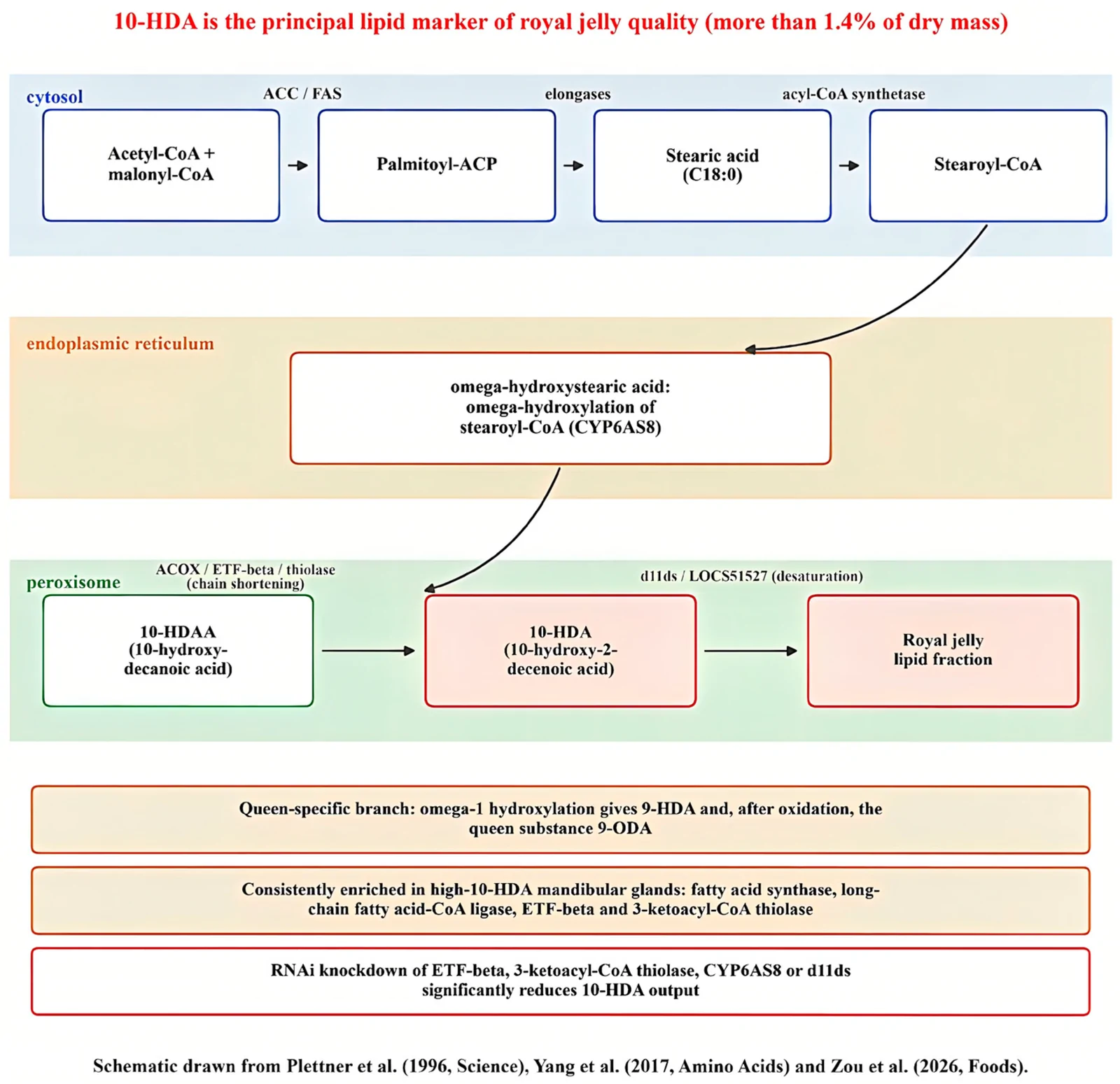
Three-stage pathway of 10-HDA biosynthesis in the worker mandibular gland: fatty-acid synthesis, omega-hydroxylation, peroxisomal beta-oxidation and desaturation [23,24,25]. Figure drawn de novo for this review has no graphical reconstruction that unintentionally implies numerical relationships absent from the original studies.

10-HDA is a short-chain hydroxy fatty acid that accounts for more than 1.4% of the dry mass of RJ and confers much of its antimicrobial activity. Isotope labelling established that the worker gland synthesizes it from acetate, using stearic acid as the principal precursor, in a three-stage pathway: de novo fatty acid synthesis supplies stearic acid, which is activated to stearoyl-CoA and omega-hydroxylated; the 18-carbon hydroxy acid is then shortened by peroxisomal beta-oxidation; and the resulting 10-carbon intermediate is desaturated to 10-HDA (Figure 5) [23,24]. Caste determines which branch is taken. Worker glands hydroxylate stearic acid at the omega position to give 10-HDA, whereas queen glands use omega-1 hydroxylation to give 9-HDA and, after oxidation, the queen substance 9-ODA [23].

### 4.2. Enzymes and the Evidence of Selection

Comparative proteomics and functional tests converge on the enzymes of the fatty-acid pathway. Proteomics identified 59 up-regulated protein species representing 45 unique proteins, spanning carbohydrate and energy metabolism, fatty-acid metabolism, protein folding, antioxidation and the cytoskeleton; within the fatty-acid group, fatty-acid synthase, long-chain fatty-acid-CoA ligase, ETF-beta and 3-ketoacyl-CoA thiolase are consistently enriched in high-10-HDA glands [24,25]. RNA interference against ETF-beta or the thiolase reduces 10-HDA accumulation, and knockdown of *CYP6AS8*, implicated in hydroxylation, or the desaturase-like gene *d11ds*/*LOC551527* lowers 10-HDA levels [24,25].

Regulation also operates above the level of enzyme abundance. A phosphoproteomic comparison of mandibular glands from the two stocks identified a distinct set of differentially phosphorylated proteins, indicating that post-translational modification contributes to the output difference [26]. Diet matters too: oleic acid supplementation remodels gland triacylglycerols and raises *CYP6AS8* and fatty-acid synthase expression, increasing 10-HDA output [24].

One caveat deserves emphasis. The mandibular contribution appears less uniformly co-selected than the hypopharyngeal one, because strain comparisons do not always rank high-yield colonies highest for 10-HDA content. The lipid pathway may therefore respond partly to diet rather than to genotype alone [3]. The identified enzymes involved in the biochemical pathway and differences in enzyme abundance provide potential candidate targets for downstream validation such as functional knockdown.

## 5. Olfaction and the Recognition of Larval Signals

A colony can only provision larvae that its nurses find and accept, which places chemosensory recognition upstream of all the processes described here.

### 5.1. Larval Pheromones as the Trigger for Nursing

Larvae solicit care chemically. The classical brood pheromone is a blend of methyl and ethyl esters of five fatty acids whose concentration rises as larvae grow and peaks at capping, while young larvae also emit beta-ocimene, which suppresses ovary activation and stimulates feeding and gland development [18].

### 5.2. A Volatile Larval Signal and Its Carrier Proteins

Two volatile terpenoids act as the active signal: solid-phase microextraction with liquid chromatography-mass spectrometry identified beta-ocimene and allo-ocimene as the principal volatiles released by 24–36 h larvae [27].

**Figure 6:**
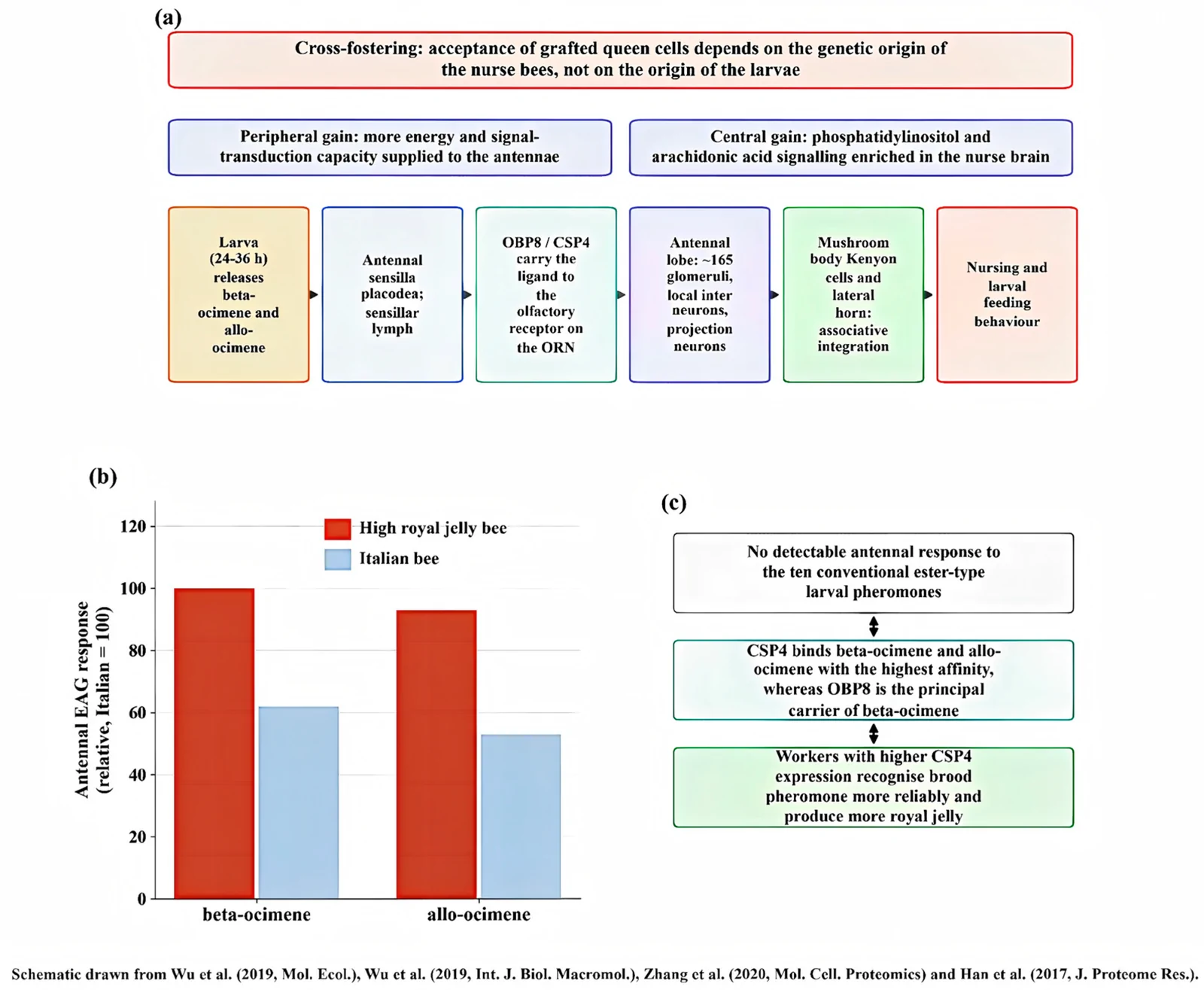
(**a**) Route from larval volatiles to nursing behaviour, including the cross-fostering result. (**b**) Electroantennogram responses of nurses of the two stocks. (**c**) Carrier proteins and their ligands [6,22,27,28]. Figure drawn de novo for this review has no graphical reconstruction that unintentionally implies numerical relationships absent from the original studies.

The difference in response to volatile larval signals in high- and low-yield RJ stocks lies in the receiver, rather than in the signal. This is indicated by electroantennogram recordings that the ITB nurse antennae do not respond detectably to the ten conventional ester-type larval pheromones, but that antennae of RJB nurses responded significantly more strongly to beta-ocimene and allo-ocimene than those of ITB nurses (Figure 6b). Cross-fostering, in turn, confirms that acceptance of grafted queen cells depends on the nurse genotype and not on the larval genotype (Figure 6a) [27].

Two carrier proteins mediate the response. Among the honeybee odorant-binding proteins (OBPs) and chemosensory proteins (CSPs), CSP4 has the highest affinity for beta-ocimene and allo-ocimene, an interaction characterized by fluorescence quenching, isothermal titration calorimetry, molecular docking and site-directed mutagenesis, while OBP8 is reported as a principal carrier of beta-ocimene [28]. Workers with higher CSP4 expression recognize brood pheromone more reliably and produce more RJ, linking carrier abundance directly to colony output (Figure 6c) [3,28]. These carriers belong to a small family: the honeybee genome encodes only 21 OBPs and six CSPs, far fewer than dipteran genomes [29]. The role of the two carrier proteins still requires functional confirmation to reveal the causality linking to high RJ syndrome.

### 5.3. Central Processing and the Conversion of Detection into Acceptance

Olfactory information travels from the antennal sensilla to roughly 165 glomeruli of the antennal lobe, then through projection neurons to the mushroom bodies and lateral horn [16].

Behavioural testing attributes the difference to detection and salience rather than learning. In proboscis-extension assays, RJB nurses responded spontaneously to larval odours more often than ITB nurses but learned at similar rates [22]. Consistently, the antennal lobe proteome of young RJBs is enriched in energy and nutrient metabolism and in signal transduction, priming the lobe to receive brood signals [22]. The antennal lobe proteome signature of young RJBs suggests that central amplification may then convert detection into acceptance. In the proteome of nurse brains of the RJBs, phosphatidylinositol and arachidonic acid pathways are enriched, which would be expected to strengthen the olfactory response to larval pheromone [30]. Higher peripheral sensitivity combined with central gain thus lets nurses identify grafted 24–36 h larvae earlier and more reliably, raising acceptance and the number of cells entering the secretion cycle (Table 1) [22,27].

**Table 1:** Tissue-resolved molecular evidence for the high-yield royal jelly syndrome by comparing high royal jelly-producing bees with unselected Italian bees.

Tissue or Target	Research Approach	Principal Findings in RJBs	Ref. and Evidence Source
Hypopharyngeal gland	In-depth proteome across the lifespan	Protein synthesis and energy metabolism induced in nurses; RpL13, RpL28, RpS4, RpS26, hexamerin 110, MDH, UGP2, citrate synthase, enolase	[19] proteome enrichment
Hypopharyngeal gland	Morphometry and proteome	Acinar diameter larger at all ages; about fivefold higher yield; size correlated with yield	[5] phenotypic assay
Hypopharyngeal gland	Proteome of age-dependent development	Age-dependent change in MRJP form and abundance tracks the shift from nursing to foraging	[5,17] proteome enrichment
Mandibular gland	Comparative proteome by 10-HDA output	59 up-regulated protein species (45 unique proteins); fatty-acid synthase and beta-oxidation enzymes implicated	[25] proteome enrichment and phenotypic assay
Mandibular gland	Phosphoproteome	Differentially phosphorylated proteins distinguish the selected stock	[26] differential protein phosphorylation
Brain	Quantitative neuropeptidome, four stages	158 neuropeptides, 77 newly reported; nurse enrichment of tachykinin, diuretic hormone and periviscerokinin; forager enrichment of sNPF, PDH, FMRFamide and corazonin	[6] differential quantitative neuropeptidome analysis
Brain	Membrane proteome and phosphoproteome	More membrane proteins at every adult stage; phosphatidylinositol and arachidonic acid signalling enriched in nurses	[30] Membrane proteome and phosphoproteome enrichment
Antenna and antennal lobe	Electroantennography, binding assays, brain proteome	Stronger response to beta-ocimene and allo-ocimene; CSP4 and OBP8 as principal carriers	[22,27,28] Electroantennography, binding assays, brain proteome enrichment
Mitochondria	Whole mitogenome resequencing	Selected-stock-dominant sublineage six times more frequent, attributable to drift; copy number unrelated to yield	[13] Whole mitogenome resequencing

## 6. Neuropeptides, Membrane Proteins and Central Regulation of Nursing and Foraging

RJ secretion is as much a behavioural trait as it is a product of glandular physiology, which makes the brain part of the phenotype rather than a bystander to the process.

### 6.1. The Neuropeptidome of Nurse and Forager Brains

Quantitative neuropeptidomics across four stages of the division of labour identified 158 non-redundant neuropeptides in the worker brain, 77 of them newly reported, substantially expanding the known honeybee neuropeptidome [6]. Fourteen precursor proteins change in the same way with age in both honeybee stocks, suggesting they are general regulators of task transition, not stock-specific features [6].

**Figure 7:**
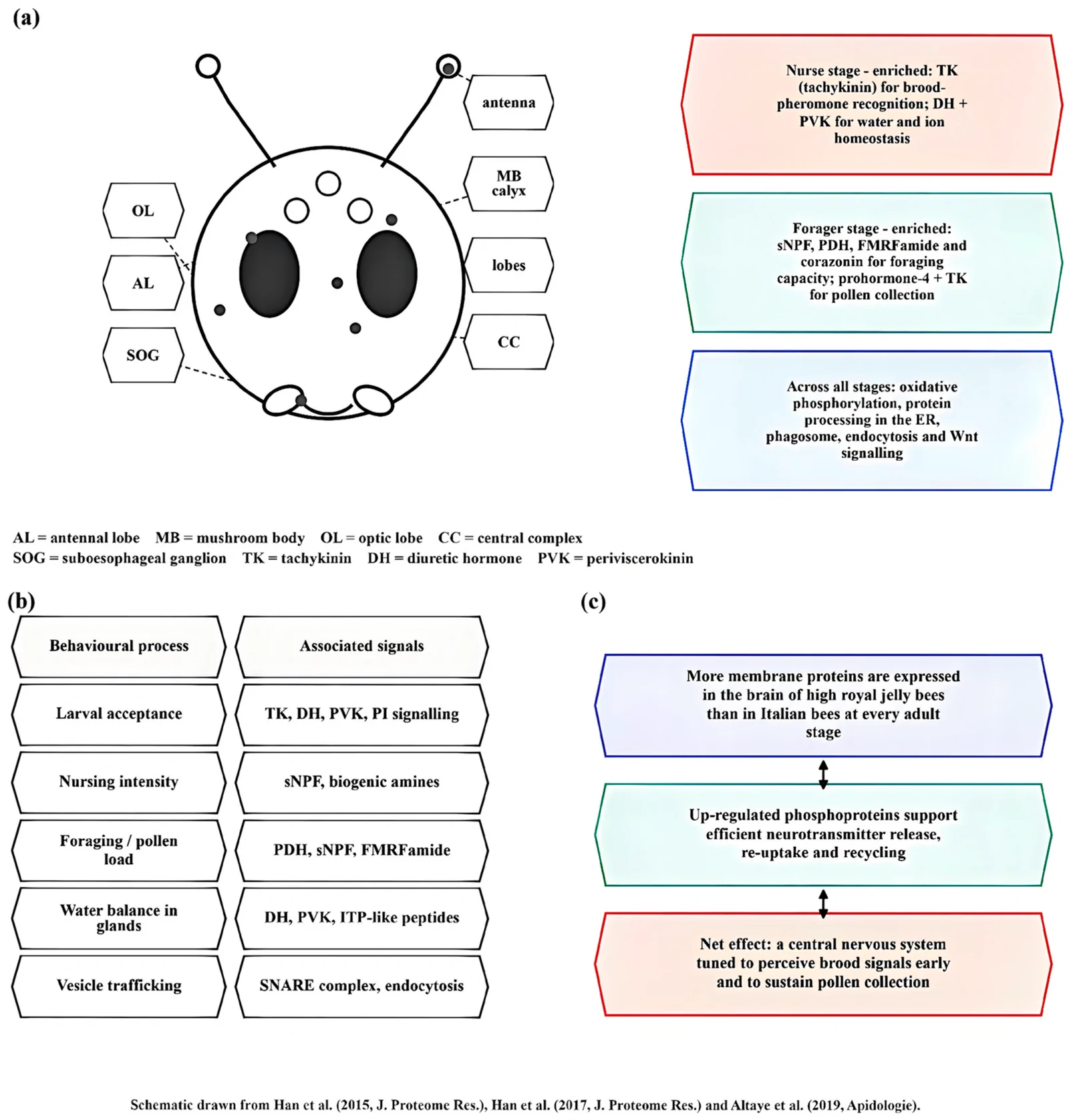
(**a**) Brain regions and the neuropeptides enriched at nurse and forager stages. (**b**) Behavioural processes mapped to signalling molecules. (**c**) Membrane-protein and phosphoprotein findings [3,6,30]. Figure drawn de novo for this review has no graphical reconstruction that unintentionally implies numerical relationships absent from the original studies.

Stock-specific differences between the two honeybee lines cluster around water homeostasis and brood recognition. In RJB nurses, the highly abundant neuropeptides governing water and ion homeostasis, namely diuretic hormone and periviscerokinin, are enriched, together with tachykinin, suggesting their roles are associated with brood pheromone recognition (Figure 7a,b) [6]. The enriched signalling presumably serves the need that the RJBs demand correspondingly larger water and ion transport to sustain the secretion of ten times more fresh RJ that contains roughly 60–70% water compared to the ITBs [6]. In foragers, the enriched peptides are likely correlated to the role of supporting food collection: sNPF, PDH, FMRFamide and corazonin are associated with foraging capacity, while prohormone-4 and tachykinin may link to pollen collection [6].

### 6.2. The Brain Membrane Proteome and Phosphoproteome

Membrane proteins and their phosphorylation states determine how neurons receive, transmit and recycle signals, and more membrane proteins are expressed in RJB brains than in ITB brains at every adult stage [30]. The enriched pathways point to accelerated vesicular transport and signal recycling. Several are shared across stages (oxidative phosphorylation, endoplasmic-reticulum protein processing, phagosome, endocytosis, Wnt signalling), whereas others are stage-specific, such as lysosomal pathways in nurses and foragers [30]. Up-regulation of SNARE interactions and endocytic machinery would be expected to accelerate the release and re-uptake of signalling molecules, raising nerve sensitivity [3,30].

Phosphorylation reinforces the same direction of change: up-regulated phosphoproteins are enriched for neurotransmitter transmission and recycling, which should support larval feeding by nurses and food collection by foragers [30]. In RJBs, nurse brains were specifically enriched in phosphatidylinositol signalling and arachidonic acid metabolism, while forager brains were enriched in signal-processing pathways that may be associated with pollen collection (Figure 7c) [30]. These omics data are useful for understanding the molecular basis of how the central nervous system is implicated in the high-yield RJ syndrome. However, in-depth biological validation is still required.

### 6.3. Biogenic Amines and Endocrine Context

Biogenic amines in the honeybee brain modulate arousal, sensory gain and task allocation. Octopamine is arousing, increases sensory sensitivity, improves learning and promotes foraging, whereas dopamine and serotonin often act antagonistically [31]. Octopamine also acts directly on the gland: topical treatment shrinks the hypopharyngeal gland, up-regulates autophagic gene expression and releases lipids into the haemolymph, identifying the gland as an energy store that can be mobilized under stress [32]. If octopamine mobilizes gland reserves and initiates the transition to foraging, then the extended secretory window observed in RJBs may result from a delayed or attenuated octopaminergic signal [32].

Endocrine and nutrient signalling provide the broader context. The vitellogenin-juvenile hormone module regulates the nurse-to-forager transition and acts upstream of insulin-like peptide 1, the adipokinetic hormone receptor, and the foraging gene *Amfor*, linking pollen intake to gland performance [33]. Overall, existing neural data indicate that the nervous system is tuned to detect brood signals earlier and more reliably, delay the switch to foraging, and allow the head’s water and energy budget to support prolonged secretion [6,22,30].

## 7. An Integrated Model of the High-Yield Syndrome

The evidence based on phenotypic and omics data reviewed above organizes into three proposed coupled loops, not a list of independent traits. The first is a recognition loop: larval volatiles could be detected by antennal carrier proteins and processed centrally, leading to acceptance and provisioning, which in turn increases brood signalling [27,28]. The second is a capacity loop: feeding behaviour activates the hypopharyngeal gland, which may enhance protein synthesis, energy metabolism and vesicle loading, enlarging acini and prolonging the competent phase, and thereby increasing secretion per accepted cell [5,19]. The third is a supply loop: forager neuropeptides and brain signalling pathways raise pollen collection and so the protein supply to nurse bees, closing the circuit that sustains secretion [6,30].

Selection has shifted the potential operating point of all three loops at once. Nothing in this model requires a new organ, a new biosynthetic route or a new sensory modality; the difference lies in thresholds, gains and durations (Figure 1). The framing also predicts trade-offs, since shifting reproductive investment upward increases the number of queens raised and would be expected to alter life-history allocation more broadly [27]. However, the proposed effects on reproductive investment, longevity, energetic allocation, and colony fitness are testable predictions.

## 8. Conclusions and Research Perspectives

RJBs are now reasonably well described at four levels that are associated with the high-yield syndrome: heritable polygenic variation, enhanced olfactory detection of larval volatiles, enlarged and longer-active hypopharyngeal glands with reinforced protein synthesis, and a remodelled brain neuropeptidome and membrane proteome.

Several questions remain open. The structure and copy number of the *mrjp* cluster have not been resolved by long-read sequencing in the selected stock, and whether the cluster itself varies between stocks is untested [15]. The causal contribution of the chromosome-scale inversion is likewise unknown, and resolving it requires association mapping at the sample sizes that simulation work indicates are necessary [4,14]. Three further gaps stand out: the olfactory receptors that read beta-ocimene and allo-ocimene have not been identified in the context of selection, the mandibular pathway is not uniformly co-selected, and the epigenomic basis of the phenotype remains largely unexplored [24,28].

Practical translation is already within reach. Potential future applications of cryopreservation of the genetic resource, marker-assisted selection using gland length, microsatellite alleles, *mrjp3* polymorphism and CSP4 or OBP8 expression, and genomic selection for the composite trait would all benefit from the evidence summarized here [3,7,11]. The broader lesson is that a large agricultural selection response in a eusocial insect can arise without obvious genomic novelty, through coordinated shifts in sensory thresholds, glandular capacity and behavioural timing.

## 9. Limitations of Current Evidence and Experimental Gaps

Although current evidence proposed three feedback loops for the high-yield syndrome of RJ, the experimental gaps remain. That is, the limited numbers of independent RJB populations; potential founder effects; differences in management conditions; colony-level rather than individual-level phenotypes; queen polyandry; environmental effects; lack of replicated functional genetic experiments; limited validation of candidate markers; difficulty separating cause from consequence in omics studies; and the absence of large-scale independent validation populations.

## Data Availability

No new data were created or analyzed in this study. Data sharing is not applicable to this article.
